# RNA-cDNA hybrids mediate transposition via different mechanisms

**DOI:** 10.1038/s41598-020-73018-y

**Published:** 2020-09-29

**Authors:** Lauren A. Todd, Amanda C. Hall, Violena Pietrobon, Janet N. Y. Chan, Guillaume Laflamme, Karim Mekhail

**Affiliations:** 1 Department of Laboratory Medicine and Pathobiology, Faculty of Medicine, University of Toronto, Toronto, ON M5G 1M1 Canada; 2 Faculty of Medicine, Canada Research Chairs Program, University of Toronto, Toronto, ON M5G 1M1 Canada

**Keywords:** DNA recombination, Molecular biology, Eukaryote

## Abstract

Retrotransposons can represent half of eukaryotic genomes. Retrotransposon dysregulation destabilizes genomes and has been linked to various human diseases. Emerging regulators of retromobility include RNA–DNA hybrid-containing structures known as R-loops. Accumulation of these structures at the transposons of yeast 1 (Ty1) elements has been shown to increase Ty1 retromobility through an unknown mechanism. Here, via a targeted genetic screen, we identified the *rnh1Δ rad27Δ* yeast mutant, which lacked both the Ty1 inhibitor Rad27 and the RNA–DNA hybrid suppressor Rnh1. The mutant exhibited elevated levels of Ty1 cDNA-associated RNA–DNA hybrids that promoted Ty1 mobility. Moreover, in this *rnh1Δ rad27Δ* mutant, but not in the double RNase H mutant *rnh1Δ rnh201Δ*, RNA–DNA hybrids preferentially existed as duplex nucleic acid structures and increased Ty1 mobility in a Rad52-dependent manner. The data indicate that in cells lacking RNA–DNA hybrid and Ty1 repressors, elevated levels of RNA-cDNA hybrids, which are associated with duplex nucleic acid structures, boost Ty1 mobility via a Rad52-dependent mechanism. In contrast, in cells lacking RNA–DNA hybrid repressors alone, elevated levels of RNA-cDNA hybrids, which are associated with triplex nucleic acid structures, boost Ty1 mobility via a Rad52-independent process. We propose that duplex and triplex RNA–DNA hybrids promote transposon mobility via Rad52-dependent or -independent mechanisms.

## Introduction

Transposable elements are repetitive DNA sequences that are widely distributed in eukaryotic genomes and can move around the genome in a copy-and-paste (Class I: retrotransposons) or cut-and-paste (Class II: DNA transposons) manner^1,2^. Retrotransposons are further divided based on the presence of flanking long terminal repeats (LTRs) such as in the transposons of yeast 1 (Ty1) elements of budding yeast and the non-LTR long interspersed nuclear elements (LINE-1) in humans^3–5^. Both Ty1 and LINE-1 elements are autonomous and have two open reading frames (TYA/TYB and ORF1/ORF2, respectively) that code for the proteins required for retrotransposition^2^. Retromobility of both Ty1 and LINE-1 begins with transcription by RNA polymerase II (RNA Pol II) – the resulting transcripts serve as template for both translation into retrotransposition proteins and reverse transcription into double-stranded complementary deoxyribonucleic acid (cDNA) that is integrated/inserted into new genomic loci. Retrotransposons account for approximately 3% and 45% of the yeast and human genomes respectively, and replicate with each round of transposition^3,6^. The hyperactivation of retromobility can compromise genome stability and is associated with various diseases including cancer and neurodegenerative diseases^7–16^.

Retrotransposons can elicit gross chromosomal rearrangements and genetic mutations that alter gene expression and ultimately give rise to an array of diseases. Although LINE-1 is the only active autonomous retrotransposon in human cells, the proteins encoded by LINE-1 act *in trans* and can promote not only the transposition of LINE-1 itself, but also that of other retrotransposons such as *Arthrobacter luteus (Alu)* elements, of which there are over one million copies in the human genome^6,17^. These elements are implicated in both somatic and germline mutations^7–16^. Additionally, the expression of LINE-1 proteins is a hallmark of many high-grade malignant cancers^18^.

A potential mechanism linked to retromobility involves RNA–DNA hybrid structures called R-loops^19,20^. R-loops are three-stranded nucleic acid structures composed of an RNA–DNA hybrid and a displaced single-stranded DNA^21^. Although R-loops are common byproducts of transcription, their aberrant accumulation can interfere with replication fork progression, triggering DNA double strand breaks (DSBs). Such breaks can be repaired by homologous recombination (HR), which at interspersed repetitive loci can give rise to aberrant chromosome rearrangements via non-allelic recombination^21,22^. In fact, the aberrant accumulation of RNA–DNA hybrids is implicated in a number of diseases including several cancers^23–26^.

Recent studies have shown that RNA–DNA hybrids accumulate at Ty1 retroelements in the absence of RNase H enzymes, which degrade the RNA component of RNA–DNA hybrids^19,20^. Studies conducted in the budding yeast *Saccharomyces cerevisiae* have shown that Rnh1 (yeast orthologue of mammalian RNase H1) detects and resolves R-loops in response to R-loop-induced stress in a cell-cycle-independent manner, while the catalytic subunit of RNase H2 (Rnh201), is only weakly associated with R-loops, resolving them in a cell-cycle-dependent manner^27,28^. Interestingly, in the absence of both Rnh1 and Rnh201, RNA–DNA hybrid accumulation at Ty1 increases its transposition via an unknown mechanism^20^.

Here, we aimed to address the gap in knowledge regarding RNA–DNA hybrid-mediated retromobility by identifying the general mechanism through which RNA–DNA hybrids increase Ty1 retromobility. We also aimed to assess if the loss of factors that repress Ty1 retromobility, in cells lacking RNA–DNA hybrid suppressors, has an effect on retromobility. We screened strains lacking RNA–DNA hybrid and retromobility suppressors as single and double mutants and identified a double mutant, *rnh1*∆ *rad27*∆, that showed both an increase in RNA–DNA hybrid accumulation at Ty1 cDNA and an exacerbated increase in Ty1 retromobility relative to cells lacking either Ty1 suppressors (*rad27*∆) or RNA–DNA hybrid suppressors (*rnh1*∆ *rnh201*∆) alone. We found that RNA-cDNA hybrids at Ty1 activate mobility through two different mechanisms. Specifically, in the *rnh1*∆ *rad27*∆ mutant, the accumulating RNA–DNA hybrids preferentially exist as a duplex nucleic acid structure and trigger Rad52-dependent mobility. In contrast, in the *rnh1*∆ *rnh201*∆ mutant, the accumulating RNA–DNA hybrids are preferentially part of *bona fide* triplex R-loop structures and activate Rad52-independent retromobility. Taken together, we find that RNA-cDNA hybrid-mediated mobilization of Ty1 in cells lacking both Ty1 and RNA–DNA hybrid regulators appears to be dependent on Rad52. In contrast, in cells lacking only RNA–DNA hybrid regulators, RNA-cDNA hybrids mobilize Ty1 through a Rad52-independent mechanism. This suggests that Ty1-associated RNA–DNA hybrids can mobilize retroelements through canonical integration or HR.

## Results

### Mutants with elevated Ty1 RNA–DNA hybrids

**Figure 1:**
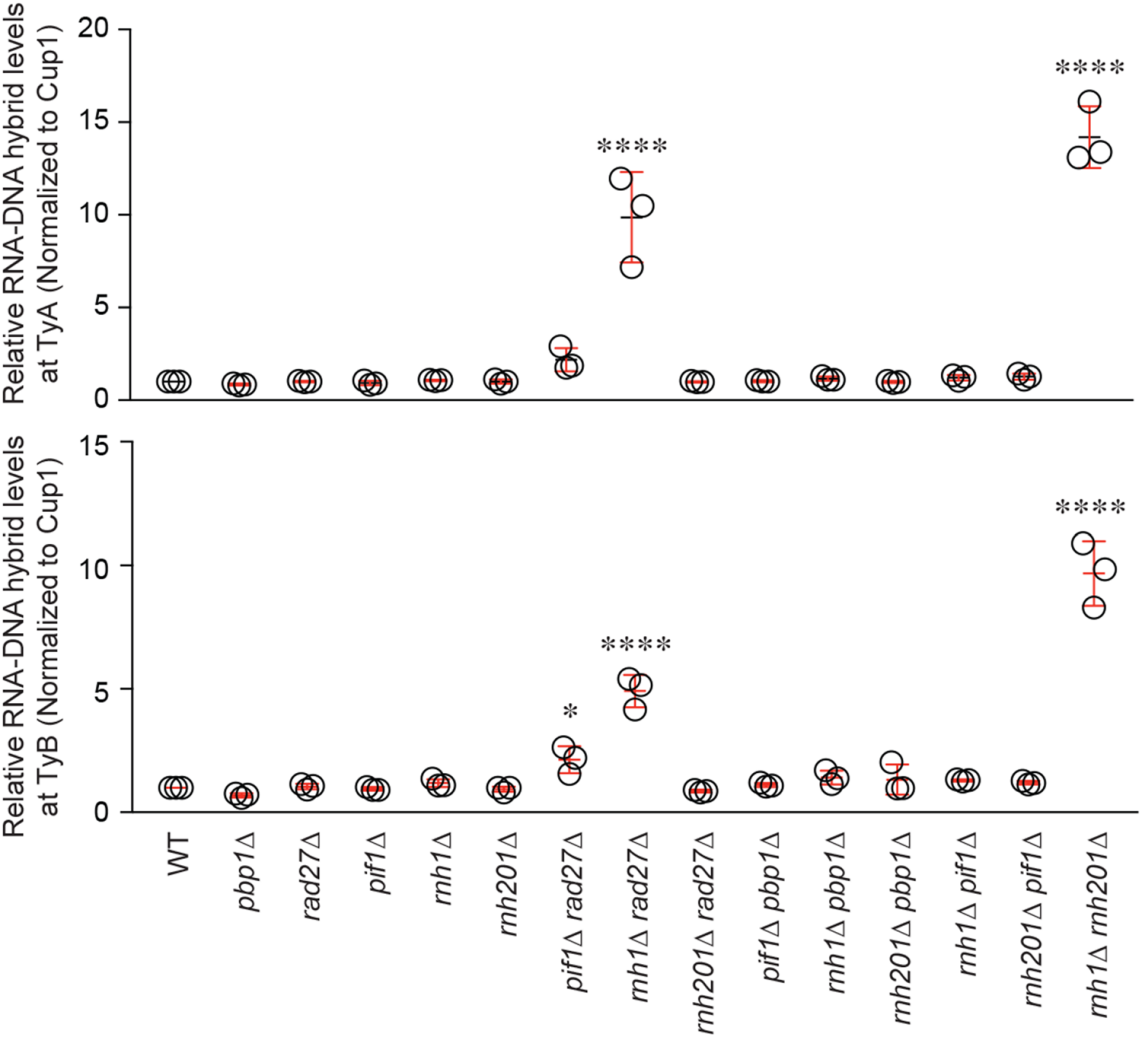
Mutants with elevated levels of Ty1 RNA–DNA hybrids. ChIP assessing the levels of RNA–DNA hybrids at Ty1 using the S9.6 antibody, followed by qPCR of *TYA* (top) and *TYB* (bottom) regions. Results are calculated as % input normalized to *CUP1* and presented relative to wild-type. (Mean ± SD; *n* = 3; one-way ANOVA followed by Dunnett’s post-hoc test). *p < 0.05; ****p < 0.0001.

**Figure 2:**
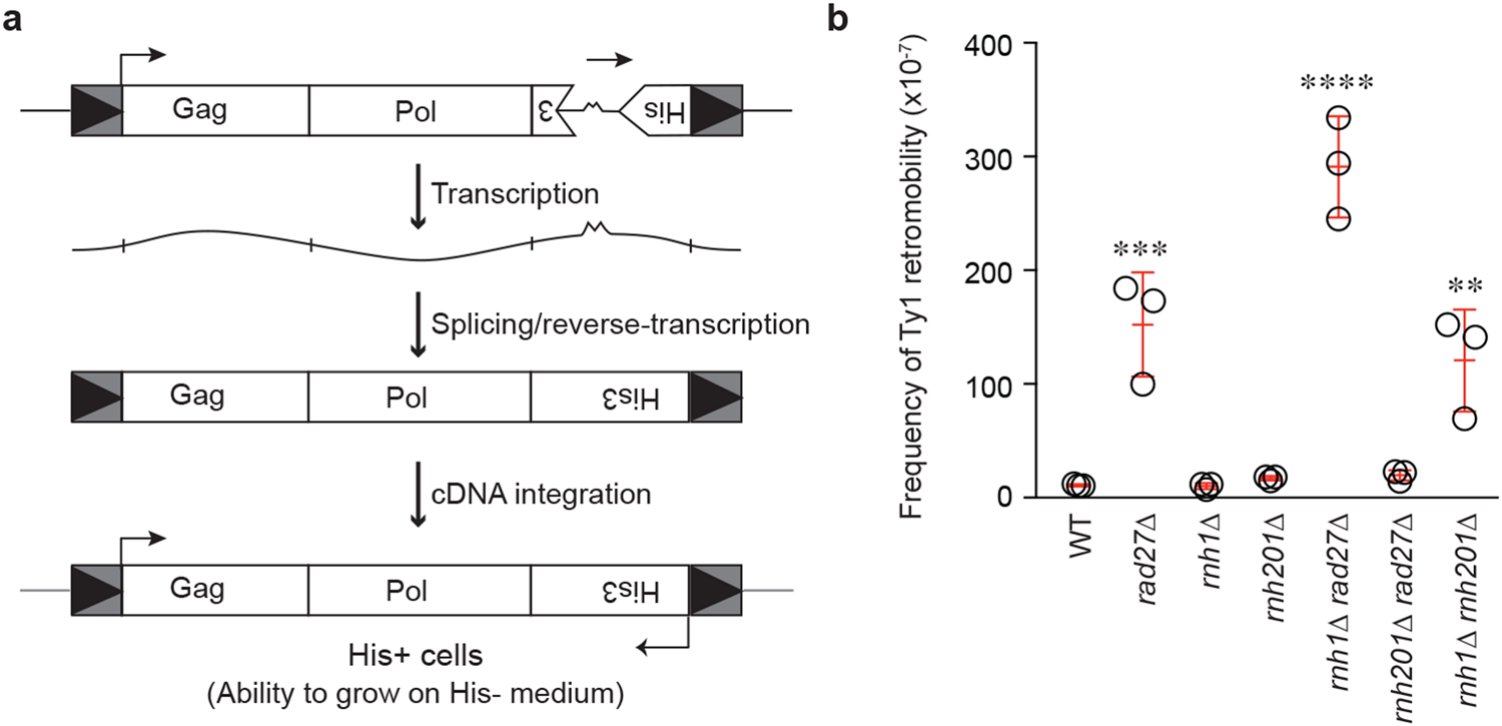
Mutants with elevated levels of Ty1 mobility. (**a**) Schematic of the *Ty1his3AI* reporter-based method to quantify Ty1 retromobility. A retromobility event converts His^−^ cells to His^+^ cells allowing for cellular growth on His-negative medium. **(b)** *Ty1his3AI* retromobility screen to identify mutants with increased Ty1 retromobility. (Mean ± SD; *n* = 3; one-way ANOVA followed by Dunnett’s post-hoc test). **(a,b)** **p < 0.01; ***p < 0.001; ****p < 0.0001.

Retromobility, which can be detrimental to genome stability, is typically repressed by various mechanisms^2^. For example, Rad27 (radiation sensitive 27) represses Ty1 retromobility by destabilizing Ty1 cDNA, and it has been suggested that Rad27 also suppresses Ty1 cDNA multimers that can promote recombination between Ty1 cDNA and genomic elements^4,29^. Additionally, RNase H enzymes limit Ty1 mobility by lowering its associated RNA–DNA hybrid levels^19,20^. In order to more broadly assess the putative role of RNA–DNA hybrid suppression at Ty1, we conducted a targeted yeast genetic screen employing single and double gene knockouts of RNA–DNA hybrid regulators (DNA or RNA-binding proteins) and/or the Ty1 inhibitor Rad27. We included knockouts of the RNase H enzymes that directly remove hybrids, the Pif1 helicase that can destabilize hybrids by acting on various DNA or RNA components of hybrid-containing structures, and the RNA-binding protein Pbp1, which binds RNA hindering its hybrid-forming potential. We note that these combinations do not represent the great diversity observed within the rapidly growing molecular network of RNA–DNA hybrid regulators. First, we assessed RNA–DNA hybrid levels at Ty1 in these mutants using chromatin immunoprecipitation (ChIP) followed by qPCR. The ChIP employed the anti-RNA–DNA hybrid antibody S9.6, which detects RNA–DNA hybrids in a sequence-independent manner, yields signals sensitive to RNase H1, and does not detect double-stranded RNA molecules using dot blot analysis (Fig. S1a,b). We identified a novel mutant, *rnh1Δ* *rad27Δ*, which showed Ty1 RNA–DNA hybrid accumulations that are comparable to the RNA–DNA hybrid build-ups in the *rnh1*∆ *rnh201*∆ control mutant (Fig. 1). Of note, *rad27Δ* or *rnh1Δ* single mutants did not exhibit elevated RNA–DNA hybrid levels. Next, we assessed the frequency of Ty1 retromobility in the *rnh1Δ* *rad27Δ* strain using the *Ty1his3AI* reporter system (Fig. 2a) ^30^. In cells lacking a functional endogenous *HIS3* gene, a synthetic *HIS3* gene containing an inverted artificial intron is placed at the end of the *TYB* open reading frame of a single Ty1 element, in the reverse orientation to Ty1. A functional *HIS3* gene is only present when Ty1 is transcribed, spliced, reverse-transcribed and the resulting cDNA is integrated into the genome allowing cells to grow on His-negative media. Using this assay, we observed that *rnh1Δ rad27Δ* cells exhibited a greatly elevated rate of retromobility when compared to wild-type cells (Fig. 2b). In fact, this elevated retromobility appeared to reflect a synergistic effect of combining the loss of Rad27 with the loss of Rnh1. In addition, the rate of Ty1 retromobility in *rnh1Δ rad27Δ* cells was also higher than the rate observed in *rad27*∆ cells or *rnh1*∆ *rnh201*∆ cells (Fig. 2b). Thus, in addition to the known *rnh1*∆ *rnh201*∆ mutant, we have identified a novel mutant, *rnh1Δ* *rad27Δ* that exhibits RNA–DNA hybrid accumulation at Ty1. We note that the level of retromobility observed in the *rnh1Δ rad27 Δ* cells and *rnh1*∆ *rnh201*∆ cells does not perfectly correlate with the magnitude of RNA–DNA hybrid accumulation. This may reflect different mechanisms driving retromobility in these mutants. Additionally, loss of the Rnh1 RNA–DNA hybrid suppressor together with the loss of the Rad27 Ty1 suppressor appeared to have a synergistic effect on Ty1 retromobility. Of note, unlike the *rnh1Δ rad27Δ* cells, the *rnh201Δ* *rad27Δ* double mutant did not exhibit an increase in either RNA–DNA hybrids or retromobility (Figs. 1 and 2b). This likely reflects a higher reliance of cells on Rnh1 over Rnh201 ^27,28^. Together, the *rnh1*Δ *rad27Δ* and *rnh1*∆ *rnh201*∆ mutants provide an ideal context for identifying the mechanism by which RNA–DNA hybrids may induce Ty1 retromobility.

### Roles for RNA-cDNA hybrids in retromobility

**Figure 3:**
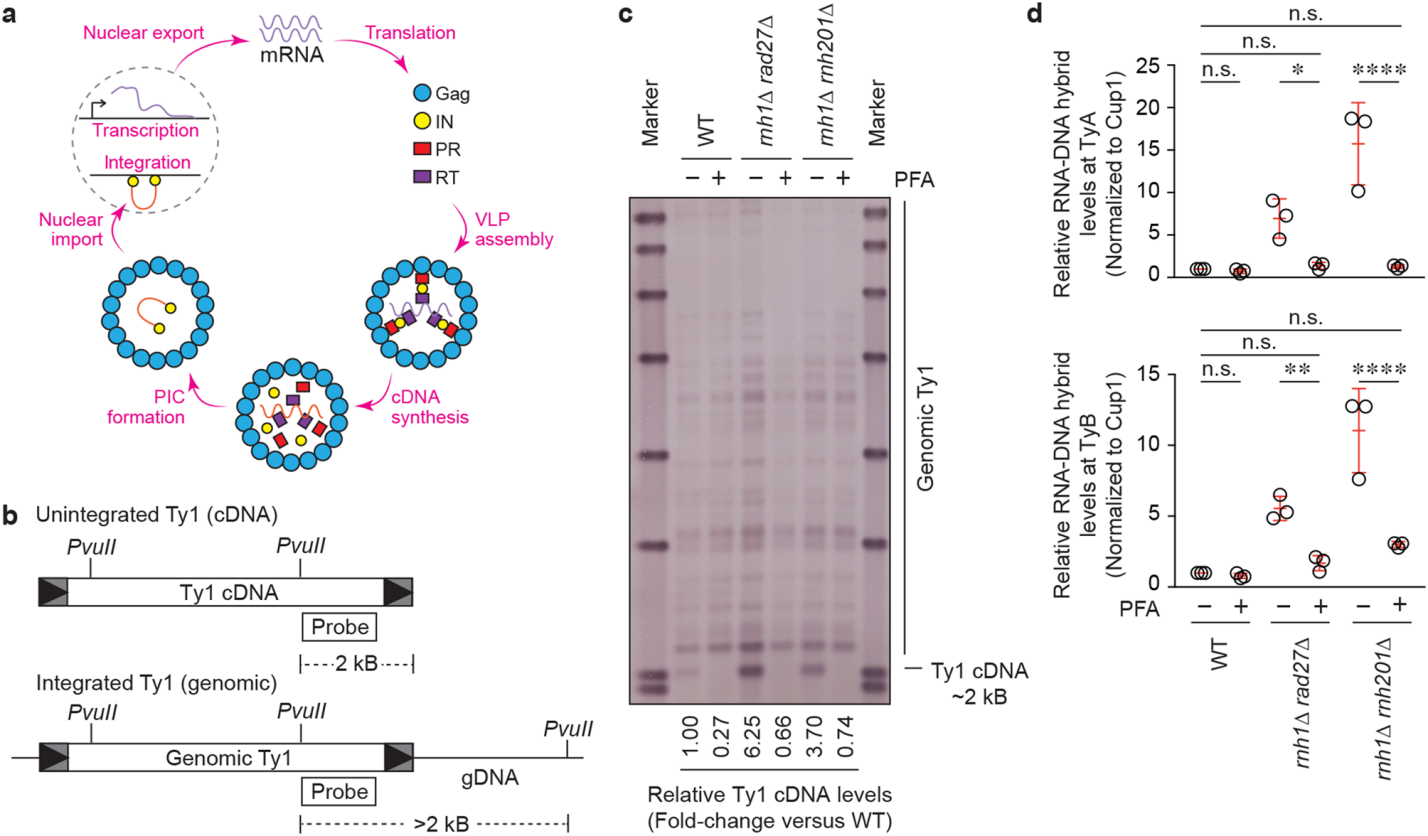
RNA–DNA hybrids accumulate on Ty1 cDNA in both *rnh1∆ rad27∆* and *rnh1∆ rnh201∆* mutants. (**a**) The Ty1 life-cycle begins with transcription of Ty1 mRNA that is both translated into the proteins required for retromobility and reverse-transcribed into cDNA which is integrated back into the genome. (**b**) A schematic of the Southern blot method used to detect and quantify Ty1 cDNA levels. (**c**) Southern blot analysis of the effect of PFA on the levels of Ty1 cDNA. (**d**) ChIP assessing the effect of PFA on levels of RNA–DNA hybrids at Ty1 using the S9.6 antibody, followed by qPCR of *TYA* (top) and *TYB* (bottom) regions. Results are calculated as % input normalized to *CUP1* and presented relative to wild-type. (Mean ± SD; *n* = 3; one-way ANOVA followed by Sidak’s post-hoc test). (**a–d**) * = *p* < 0.05; ** = *p* < 0.01; ****p < 0.0001.

During its life-cycle, Ty1 makes an identical DNA copy of itself (Ty1 cDNA), which can represent chromosomal (genomic) or extrachromosomal (cDNA) Ty1 (Fig. 3a). As such, prior to identifying the mechanism by which RNA–DNA hybrids regulate Ty1 retromobility, we asked whether the observed RNA–DNA hybrids accumulate on chromosomal Ty1 elements or on Ty1 cDNA. To test this, we chemically blocked synthesis of Ty1 cDNA using the reverse-transcriptase inhibitor phosphonoformic acid (PFA). Using Southern blotting, we confirmed that PFA treatment rendered Ty1 cDNA signals, but not genomic Ty1 control signals, undetectable in wild-type cells, *rnh1Δ* *rad27Δ* cells and *rnh1Δ* *rnh201Δ* cells (Figs. 3b,c and S2). Next, we used ChIP to assess RNA–DNA hybrid levels at Ty1 in cells treated with PFA or vehicle control. We found that RNA–DNA hybrid accumulation at both TyA and TyB decreased by 70–90% upon PFA treatment in both *rnh1Δ rad27Δ* cells and *rnh1Δ rnh201Δ* cells (Fig. 3d). In addition, apparent residual RNA–DNA hybrid levels at TyB in PFA-treated *rnh1Δ rnh201Δ* cells were not statistically significant compared to wild-type cells (Fig. 3d). This suggests that the majority of the RNA–DNA hybrids are associated with Ty1 cDNA and are therefore RNA-cDNA hybrids. This is in line with previous research indicating that Ty1-associated RNA–DNA hybrids are associated with Ty1 cDNA in the *rnh1Δ rnh201Δ* control strain^20^. However, we cannot completely rule out the possibility that low levels of RNA–DNA hybrids may also accumulate at chromosomal TyB as there exist qualitative residual RNA–DNA hybrid levels at TYB in PFA-treated *rnh1Δ rnh201Δ* cells that show no Ty1 cDNA in Southern blotting (Fig. 3c,d). Alternatively, in our PFA-treated cells, there may remain low levels of cDNA molecules that are below the detection limit of our assays. Nonetheless, the data indicate that the majority of Ty1-associated RNA–DNA hybrids in *rnh1Δ rad27Δ* cells and *rnh1Δ rnh201Δ* cells are located on Ty1 cDNA.

**Figure 4:**
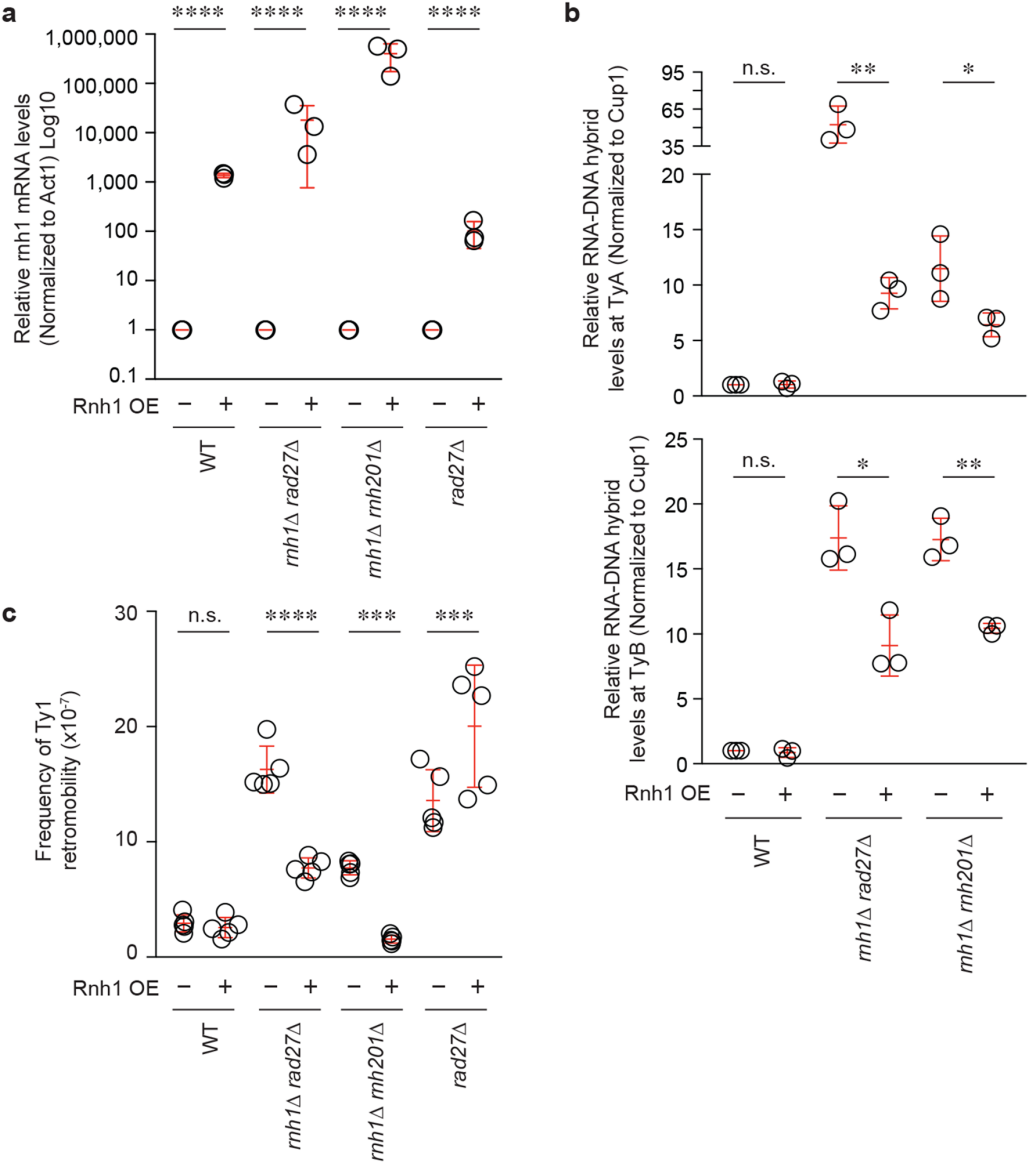
Increased Ty1 retromobility in *rnh1∆ rad27∆* and *rnh1∆ rnh201∆* is reversed upon Rnh1 overexpression. (**a**) Confirmation of Rnh1 over-expression by RT-qPCR. Results are normalized to ACT1 and are presented relative to each mutant’s empty vector control. Statistical significance was assessed by Student’s *t*-test. Mean ± SD; *n* = 3. (**b**) ChIP assessing the effect of Rnh1 over-expression on RNA-cDNA hybrids at Ty1 followed by qPCR directed at *TYA* (left) and *TYB* (right). Results are calculated as % input normalized to *CUP1* and presented relative to wild-type. Statistical significance was determined using a one-way ANOVA followed by Sidak’s post-hoc test. Mean ± SD; *n* = 3. (**c**) Effect of Rnh1 over-expression on *Ty1his3AI* retromobility. Statistical significance was assessed by a one-way ANOVA followed by Sidak’s post-hoc test. Mean ± SD; *n* = 5. (**a-c**) *p < 0.05; **p < 0.01; ****p < 0.0001.

Next, we asked if RNA-cDNA hybrid-associated increases in Ty1 retromobility can be decreased by over-expressing yeast Rnh1 (Fig. 4a). Indeed, using ChIP experiments, we found that the over-expression of Rnh1 decreased Ty1 RNA-cDNA hybrid levels and Ty1 retromobility rates in both *rnh1Δ* *rad27Δ* cells and *rnh1*∆ *rnh201∆* cells (Fig. 4b,c). In contrast, Rnh1 over-expression did not alter retromobility in *rad27Δ* cells (Fig. 4c), which do not exhibit any increases in RNA–DNA hybrid levels as compared to wild-type cells (Fig. 1). These results suggest that RNA-cDNA hybrid accumulation in *rnh1Δ* *rad27Δ* cells or *rnh1*∆ *rnh201*∆ cells promotes Ty1 mobility. Importantly, these data indicate that the RNA–DNA hybrid accumulation and its impact on retromobility in these mutants is reversible.

### Mechanisms of hybrid-mediated retromobility

**Figure 5:**
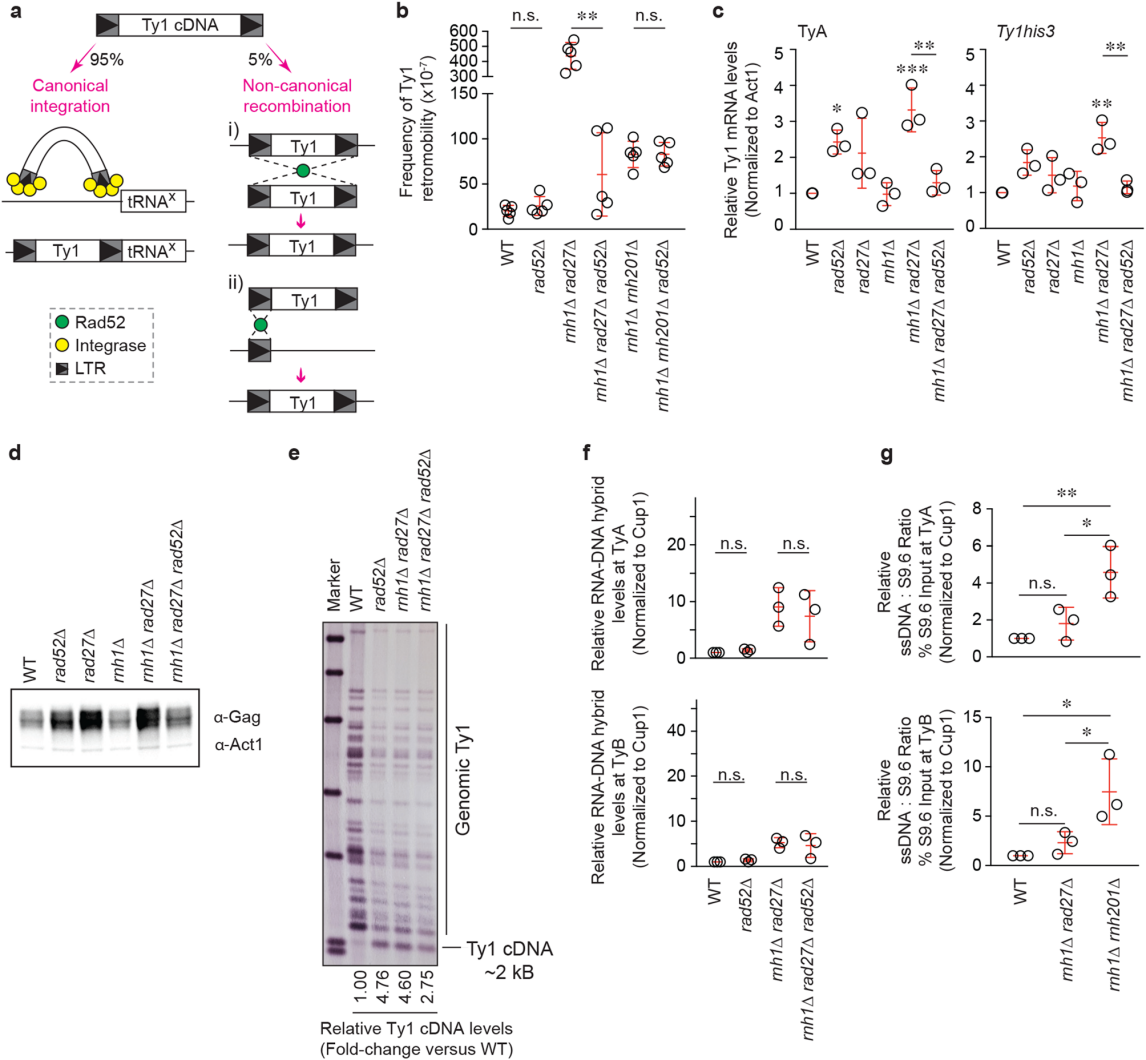
Increased Ty1 retromobility in *rnh1∆ rad27∆* but not *rnh1∆ rnh201∆* mutants is dependent on Rad52. (**a**) A schematic of canonical (integrase) and non-canonical (HR) mechanisms of Ty1 mobility. Ty1 can mobilize by HR through: (i) recombination anywhere along a Ty1 sequence, or (ii) recombination with a solo LTR. (**b**) Effect of Rad52 loss on *Ty1his3AI* retromobility (one-way ANOVA followed by Sidak’s post-hoc test). Mean ± SD; *n* = 5. (**c**) Effect of Rad52 loss on Ty1 mRNA levels (one-way ANOVA followed by Sidak’s post-hoc test). *TYA* (left) and *Tyhis3* (right) were amplified and ACT1 was used as an internal control. Mean ± SD; *n* = 3. (**d**) Effect of Rad52 loss on Ty1 Gag levels determined by western blot. Act1 is presented as a loading control. (**e**) Effect of Rad52 loss on Ty1 cDNA levels as detected by Southern blot. M = marker. (**f**) Effect of Rad52 loss on levels of RNA-cDNA hybrids at *TYA* (top) and *TYB* (bottom; one-way ANOVA followed by Sidak’s post-hoc test). Mean ± SD; *n* = 3. (**g**) Sequential ChIP assessing the co-enrichment of RNA-cDNA hybrids and ssDNA at Ty1 followed by qPCR directed at *TYA* (top) and *TYB* (bottom). Results are calculated as % S9.6 input normalized to *CUP1* and presented relative to wild-type. Statistical significance was assessed using one-way ANOVA followed by Tukey’s post-hoc test. Mean ± SD; *n* = 3. (**a–g**) *p < 0.05; **p < 0.01; n.s. = not statistically significant.

Given that RNA–DNA hybrids are known to induce recombination between repetitive DNA units ^31^, we wondered whether RNA-cDNA hybrid accumulation at Ty1 might cause Ty1 elements to move through HR rather than the canonical integration pathway (Fig. 5a). In wild-type cells under standard cell culture conditions, Ty1 predominantly integrates through canonical integration, while HR is known to mobilize Ty1 elements at a much lower rate^4,32,33^. Under certain cellular contexts, such as when integration is blocked or at higher temperatures, this distribution can be altered such that there is a preferential shift towards HR^34,35^. To test whether RNA-cDNA hybrids increase Ty1 retromobility through HR, we assessed the effect of deleting the HR factor Rad52 on Ty1 retromobility. Unexpectedly, Rad52 was required for the increase in Ty1 mobility in *rnh1Δ* *rad27Δ* cells, but not in *rnh1Δ* *rnh201Δ* cells (Fig. 5b). Therefore, we aimed to determine what effect Rad52 has on the different quantifiable Ty1 life-cycle stages, namely mRNA, Gag protein and cDNA (Fig. 3a). Levels of Ty1 mRNA, Gag and cDNA were elevated in *rnh1Δ* *rad27Δ* cells (Fig. 5c–e). Loss of Rad52 in *rnh1 Δ* *rad27Δ* cells abolished increases in the levels of Ty1 mRNA and Gag protein but only partially decreased Ty1 cDNA levels (Figs. 5c–e and S3a). In addition, the loss of Rnh1 in a *rad27Δ* background only slightly increased (~ 1.4 fold) the levels of Ty1 cDNA (Fig. S3b,c) and Rad52 deletion failed to alter RNA-cDNA hybrid levels in *rnh1Δ* *rad27Δ* cells (Fig. 5f). Of note, Rad52 loss does not completely abolish mobility in *rnh1Δ* *rad27Δ* cells (Fig. 5b). This likely reflects the fact that when RNA–DNA hybrid and thus Rad52-dependent increases in mobility are eliminated in *rnh1Δ rad27Δ* cells via Rad52 deletion, the increases in Ty1 RNA levels related to Rad27 loss remain (Fig. 5c). Overall, these data suggest that the loss of Rad52 in *rnh1Δ* *rad27Δ* cells does not directly change the levels of RNA-cDNA hybrids but rather prevents them from inducing recombination between Ty1 elements.

However, if *rnh1Δ* *rad27Δ* cells and *rnh1Δ rnh201Δ* cells both exhibit the same RNA–DNA hybrid accumulation-dependent increases in Ty1 mobility, what could underlie the observed differential dependence on Rad52? Notably, while RNA–DNA hybrids are an R-loop component, their presence is not always associated with a *bona fide* R-loop. As RNA–DNA hybrid accumulation occurs on double-stranded Ty1 cDNA in both mutant strains, we wondered if the accumulating Ty1 RNA-cDNA hybrids in *rnh1Δ rad27Δ *cells and *rnh1Δ rnh201Δ* cells may still differ structurally. For example, the RNA–DNA hybrids in only one of these two mutants may exhibit a relative preference for being part of a triplex nucleic acid R-loop structure. Therefore, we conducted a sequential ChIP (ChIP-re-ChIP) experiment in which we employed anti-RNA–DNA hybrid pulldown followed by another pulldown with an anti-single-stranded DNA antibody (anti-ssDNA). For sequential ChIP data analysis, to assess how much ssDNA is present in every unit of RNA–DNA hybrid, we divided the signal from ssDNA pulldown by the signal obtained from the anti-RNA–DNA hybrid pulldown. A mutant with duplex Ty1 RNA-cDNA hybrid structures will yield a ratio that is not statistically different from the ratio of wild-type cells, while a mutant with triplex R-loop levels at Ty1 will have a ratio that is significantly greater than wild-type ratios. Importantly, these ChIP-re-ChIP assays revealed that the ratio of ssDNA over RNA–DNA hybrids in *rnh1Δ rnh201Δ* cells was significantly higher than the ratios observed in wild-type cells or *rnh1Δ rad27Δ* cells (Fig. 5g). In contrast to the effect of *rnh1Δ rnh201Δ* double knockout at Ty1 cDNA, no significant change was observed in the relative ratios at rDNA repeats and telomeres in *rnh1Δ rnh201Δ* cells (Fig. S4). Additionally, we included in our analysis *pbp1Δ* cells, which are known to exhibit triplex R-loop accumulations at rDNA and telomeres^31,36^. As expected, despite the fact that Pbp1 prevents triplex R-loop accumulations at the rDNA and telomeric sites tested^31,36^, the loss of Pbp1 did not alter the relative ssDNA/RNA–DNA hybrid ratios in our ChIP-re-ChIP assays (Fig. S4). These results indicate that compared to wild-type cells, only *rnh1Δ rnh201Δ* cells exhibit statistically significant increases in ssDNA/RNA–DNA hybrid ratios, and this was only observed at Ty1.

**Figure 6:**
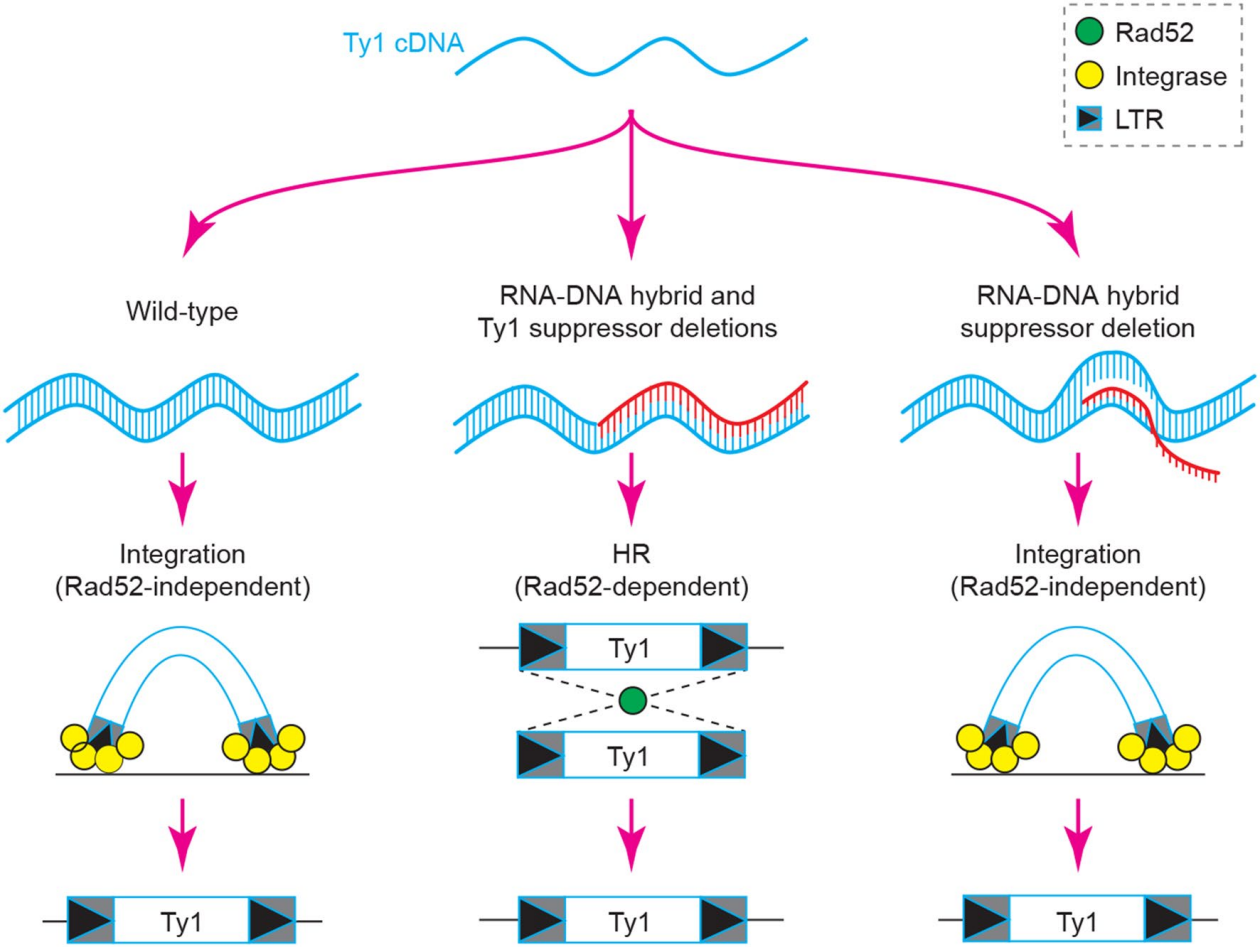
Proposed model. In wild-type cells, baseline levels of retromobility occur through the canonical integration pathway. In cells lacking both RNA–DNA hybrid and Ty1 suppressors, RNA–DNA hybrids appear to be part of a duplex nucleic acid structure and increase retromobility through the Rad52-dependent HR pathway. In contrast, in cells lacking only RNA–DNA hybrid suppressors, RNA–DNA hybrids appear to be part of triplex R-loop structures that increase retromobility through a Rad52-independent mechanism.

Taken together, our data point to a model (Fig. 6) in which RNA-cDNA hybrids may promote Ty1 mobility as part of a triplex nucleic acid-containing R-loop structure that elevates canonical integration in *rnh1Δ rnh201Δ* cells, or as part of a double-stranded RNA-cDNA duplex structure, promoting Rad52-dependent mobilization in *rnh1Δ rad27Δ *cells.

## Discussion

The loss of RNase H enzymes has been shown to increase RNA–DNA hybrid accumulation at Ty1, leading to an increase in retromobility through an unknown mechanism^19,20^. Both RNA–DNA hybrids and retrotransposons are implicated in many diseases, making it beneficial to dissect if and how these two factors may intersect. Here we examined *S. cerevisiae* deletion mutants in an attempt to identify the general process by which RNA–DNA hybrids promote Ty1 retromobility, and to assess if an interplay exists between known RNA–DNA hybrid modulators and Ty1 regulators.

Our findings suggest that RNA–DNA hybrids at Ty1 are not necessarily created equal (Fig. 6). Under certain contexts, RNA-cDNA hybrids exist as a double-stranded nucleic acid structure and may increase retrotransposon mobility through a Rad52-dependent pathway as is seen in *rnh1Δ rad27Δ *cells. In contrast, when RNA-cDNA hybrids are part of a *bona fide* R-loop structure, in the context of *rnh1Δ rnh201Δ *cells, increases in Ty1 retromobility are independent of Rad52. In wild-type cells under standard cell culture conditions, Ty1 cDNA preferentially integrates at genomic integration hotspots through the function of the Ty1 integrase protein, while only a small fraction (~ 5%) of Ty1 cDNA acts through HR^4,32,33,37^. HR between double-stranded Ty1 cDNA and genomic Ty1 sequences is dependent on Rad52, suggesting that in *rnh1Δ rad27Δ *cells, where RNA–DNA hybrid-mediated retromobility is Rad52-dependent, there is likely a preferential shift from integrase-dependent to HR-dependent Ty1 mobility ^4^. This shift to HR has previously been observed in *sir4Δ *cells, which allow for retromobility at typically non-permissive temperatures (≥ 30 °C), and when integrase activity is blocked^34,35^. Additionally, it has been shown that HR promotes the formation of Ty1 cDNA multimers^29,38^, which increase Ty1 retrotransposition. It was also found that *rrm3*∆ cells exhibit increased multimeric integration that is suppressed by Rnh1 over-expression^38^, suggesting that RNA–DNA hybrids could promote Ty1 multimer formation. However, this study did not directly assess RNA–DNA hybrid levels at Ty1 in *rrm3*∆ cells or upon Rnh1 over-expression in a *rrm3*∆ background. Thus, it is possible that Ty1-associated RNA–DNA hybrids in *rnh1Δ* *rad27Δ* cells promote Ty1 retromobility through multimers, which does not occur in *rnh1Δ* *rnh201Δ* cells since retromobility in these cells is not dependent on Rad52/HR.

Potential residual amounts of RNA–DNA hybrids observed in PFA-treated *rnh1Δ rnh201Δ* cells without detectable cDNA suggests that such remaining RNA–DNA hybrids may reflect canonical R-loop structures associated with chromosomal Ty1 elements. The observation that such residual hybrids were detected at the TyB but not TyA of these PFA-treated cells may reflect the fact that spontaneous chromosomal hybrids form more frequently near the end of genes^39,40^. Thus, in our mutant conditions, it is possible that low levels of spontaneous chromosomal R-loops are formed only at TyB, whereas non-chromosomal RNA-cDNA hybrid-containing structures can form at TyA or TyB. Future work should aim to explore whether any chromosomal hybrid levels remaining at TyB in PFA-treated *rnh1Δ rnh201Δ* cells contribute to Rad52-independent retrotransposition.

While the loss of Rad27 alone leads to increased Ty1 retromobility, we observed here that *rad27Δ* cells do not exhibit increased RNA–DNA hybrid levels at Ty1, as previously shown^29^. The synergistic impact of losing Rnh1 and Rad27 on RNA–DNA hybrid accumulation and Ty1 retromobility also suggests that the phenotypes observed in the *rnh1Δ* *rad27Δ* double mutant are not simply due to Rad27 loss. Consistent with this notion, unlike *rnh1Δ* *rad27Δ* cells, *rnh201Δ* *rad27Δ* cells do not exhibit elevated levels of Ty1 RNA–DNA hybrids or retromobility. Importantly, although the *rnh1Δ* *rad27Δ* mutant exhibits slightly elevated cDNA levels compared to the *rad27Δ* mutant, our data clearly show that the increased retromobility in *rnh1Δ* *rad27Δ* cells is driven by RNA–DNA hybrid accumulation and not simply the elevated cDNA levels. Yet, increased cDNA levels could promote RNA–DNA hybrid formation in some cellular contexts and so we cannot fully discount this possibility. We also found that RNase H1 over-expression results in a relative decrease in Ty1 retromobility in *rnh1Δ* *rad27Δ* cells but not *rad27Δ* cells, further pointing to a genetic context that is unique to *rnh1Δ* *rad27Δ* cells. This unique context of the *rnh1Δ* *rad27Δ* mutant is also reinforced by the fact that Rad52 deletion is lethal in *rad27Δ* cells but not *rnh1Δ* *rad27Δ* cells (Fig. 5)^41,42^. Taken together, our data suggest that the phenotypes observed in the *rnh1Δ* *rad27Δ* mutant cannot be attributed to the effects of losing Rad27 alone.

That different types of RNA–DNA hybrid-containing structures can promote different modes of Ty1 mobility was an unexpected finding of our study. Future studies should explore the detailed mechanistic intermediates underlying such differences. For example, we speculate that in *rnh1Δ* *rnh201Δ* cells, DNA- or RNA-binding proteins may allow Ty1 mRNA to compete for binding with its complementary DNA strand on Ty1 cDNA. This RNA could then be detached at the site of integration allowing the freed ssDNA to bind again to its complementary DNA strand, giving rise to canonical integration of Ty1 cDNA. In contrast, in *rnh1Δ* *rad27Δ* cells, we speculate that increases in Ty1 cDNA levels are paralleled by defects in Ty1 mRNA degradation following the synthesis of Ty1 cDNA (e.g. the mRNA template fails to be removed). The resulting unscheduled duplex hybrid structures may then affect integrase activity causing a shift to Rad52/HR-dependent mobility.

Our work uncovers a role for Rad52 in RNA–DNA hybrid-mediated retromobility. In *rnh1Δ rad27Δ* cells, elevated levels of RNA-cDNA hybrids, which are associated with duplex nucleic acid structures, boost Ty1 mobility via a Rad52-dependent mechanism. In contrast, in *rnh1Δ rnh201Δ* cells, elevated levels of RNA-cDNA hybrids, which are associated with triplex nucleic acid structures, boost Ty1 mobility via a Rad52-independent process. Taken together, our findings indicate that RNA–DNA hybrids may promote the mobility of transposable elements via Rad52-dependent and -independent mechanisms. As RNA–DNA hybrids and retrotransposons have already been independently linked to human disease, our findings suggest that future studies should assess if the joint dysregulation of RNA–DNA hybrid and transposable element regulators is especially detrimental in the clinical setting. More broadly, it will be also critical to determine if RNA–DNA hybrids across eukaryotic genomes exert different effects depending on the existence of such hybrids within duplex or triplex nucleic acid structures.

## Methods

### Yeast culture

Yeast strains were grown in YEPD (20 g/L peptone, 10 g/L yeast extract, 2% glucose) when antibiotic/amino acid selection was not required, or synthetic complete medium (2 g/L amino acid dropout powder, 6.67 g/L yeast nitrogen base, 2% galactose) for plasmid maintenance^36^. To induce Ty1 retromobility, precultures were grown at 30 °C and switched to 22 °C.

### Yeast strain generation

Yeast transformations were conducted using standard lithium acetate-based protocols^36,43^. Log phase cells were resuspended in LiOAC mix (100 mM lithium acetate pH 7.3, 10 mM Tris–HCl, pH 8.0, 1 mM EDTA) and incubated with transforming DNA, salmon sperm DNA (10 µg/µL) and PEG Mix (21 g PEG3350, 100 mM LiOAc, pH 7.3, 10 mM Tris–HCl, pH 8.0, 1 mM EDTA) at 30 °C for 45 min. DMSO was added and cells were heat-shocked at 42 °C for 15 min. Subsequently, cells were pelleted, resuspended in SOS mix (1 M sorbitol, 1/3 v/v YEP media, 6.5 mM CaCl_2_) and plated on antibiotic-containing YEPD or amino acid dropout plates. Deletions strains were validated by colony PCR and over-expressions were confirmed by reverse-transcription quantitative polymerase chain reaction (RT-qPCR). All yeast strains used in this study are presented in Table S1. All primers are listed in Table S2.

### Chromatin immunoprecipitation

Chromatin immunoprecipitation experiments were performed as previously described^31,36^. Yeast strains were saturated at 30 °C, and then diluted (1:4) and grown to log phase at 22 °C. Log phase cells were cross-linked for 30 min (1% formaldehyde, gentle shaking), washed (20 mM Tris–HCl, pH 7.6, and 150 mM NaCl), resuspended in lysis buffer (50 mM HEPES KOH, pH 7.5, 500 mM NaCl, 1 mM EDTA, 1% Triton X-100, 0.1% sodium deoxycholate, 0.1% SDS, 1 mM PMSF, Sigma cOmplete protease inhibitor) and lysed with glass beads. Cell lysates were sonicated (20 s at 40% amplitude; three times with 2 min intervals on ice), cleared and split into input and IP samples. S9.6 antibody (2 μg; generated in-house) was added to IP samples, which were incubated with rotation at 4 °C for 2 h. Protein-A sepharose beads were added and incubation was performed for another hour at 4 °C. The beads were then washed in lysis buffer, followed by lithium chloride buffer (10 mM Tris–HCl, pH 8, 250 mM LiCl, 0.5% NP-40, 0.5% sodium deoxycholate, 1 mM EDTA) and TE (10 mM Tris–HCl, pH 8, 1 mM EDTA) to remove unbound DNA. Bound DNA was eluted from the beads (50 mM Tris–HCl, pH 8, 10 mM EDTA, and 1% SDS). Input and IP samples were incubated at 65 °C overnight for reverse crosslinking. Samples were incubated at 37 °C for 30 min with 0.2 μg/μL RNase A, and for 2 h with 0.03 μg/μL glycogen and 0.2 μg/μL proteinase K to remove RNA and protein. DNA was isolated and analyzed by qPCR (1 cycle of 95 °C for 5 min, 60 °C for 30 s followed by 39 cycles of 95 °C for 5 s, 60 °C for 30 s and a final melt curve from 65 °C to 95 °C at 0.5 °C increments with 5 s/step). For qPCR analyses, IP signals were divided by input signals and normalized to CUP1 controls.

### Retromobility assay

Retromobility assays were performed as previously described^30,36^. Yeast strains were grown in YEPD (or SC-Ura for strains over-expressing Rnh1) overnight to saturation at 30 °C and diluted 1:1,000 into fresh medium. Diluted cultures were saturated at 22 °C (3–4 O/N). Various aliquots ranging from 100 µL to 1000 µL were plated onto SC-His (or SC-His-Ura for strains over-expressing Rnh1) plates, to determine the number of His + cells (those that have undergone a *Ty1his3AI* retromobility event). Serially diluted aliquots were plated onto SC (or SC-Ura for strains over-expressing Rnh1) control plates to quantify viable cells. Ty1 retromobility was calculated as the number of His + cells divided by the total number of viable cells plated on –His media. Where possible, two clones of each strain were assessed.

### DNA isolation

DNA isolation was performed as previously described^36,44^. Yeast strains were saturated at 30 °C, and then diluted and grown to saturation at 22 °C. Saturated cells were centrifuged and washed with sterile dH_2_O. Cells were resuspended in genomic lysis solution (2% Triton X-100, 1% SDS, 100 mM NaCl, 10 mM Tris–HCl, pH 8, 1 mM EDTA). Phenol:chloroform:isoamyl alcohol (25:24:1) and glass beads were added followed by gentle bead-beating for 4–8 min at RT. The samples were centrifuged (16,000×*g*, 4 °C, 5 min) to separate organic and inorganic phases. DNA was precipitated with 3 M NaOAc, pH 5.2 and 100% EtOH, centrifuged (16,000×*g*, 4 °C, 15 min), washed with 70% EtOH and dried. DNA was resuspended in TE (10 mM Tris–HCl, pH 8, 1 mM EDTA) and treated with RNase A (10 mg/mL) at 37 °C for 10 min.

### Southern blot

Southern blots for Ty1 cDNA were performed as previously described^45,46^ with small modifications. Yeast strains were saturated at 30 °C, and then diluted and grown to log phase at 22 °C. DNA was isolated as described above. Southern blotting was carried out using the DIG High Prime DNA Labeling and Detection Kit I (Sigma) following manufacturer’s recommended protocols. Briefly, DNA was digested with *Pvu*II and fragments were electrophoretically separated on a 1% agarose gel (30 V 16 h). DNA fragments were depurinated (0.25 M HCl), denatured (1.5 M NaCl; 0.5 M NaOH), neutralized (1.5 M NaCl; 500 mM Tris pH 7.5) and subjected to capillary transfer (20X SSC) overnight. Membranes were crosslinked, probed and developed as outlined by the kit manual. Probes were generated against Ty1 using primers Ty1 cDNA-F and Ty1 cDNA-R^20^ to produce a ~ 1.5 kb fragment, following protocols outlined in the kit manual. Uncropped blot images are included in Fig. S5.

### RNA isolation and RT-qPCR

RNA isolation and RT-qPCR were performed as previously described^36,47^. Yeast strains were saturated at 30 °C, diluted (1:4) and grown to log phase at 22 °C. Cells pellets were resuspended in AE buffer (50 mM NaOAc, pH 5 and 10 mM EDTA in 0.1% DEPC) and 10% SDS and acidic phenol (pH 4.5) were added. Samples were heat-shocked at 65 °C for 4 min, then cold-shocked for 2 min. RNA was then phenol:choloroform extracted, washed with ethanol and resuspended in DEPC-treated H_2_O. RNA was quantified (NanoDrop) and stored at − 80 °C. RNA was DNase I-treated and reverse-transcribed. A 20 µL reaction was carried out with MMLV reverse-transcriptase (Invitrogen) following manufacturer’s protocols. Reactions were carried out at 25 °C for 10 min, 37 °C for 60 min, and 70 °C for 15 min. cDNA was diluted 1:4 and qPCR was performed as described above. Results were analyzed using the ΔΔCt method (normalized to ACT1).

### Protein isolation

Protein was isolated as previously described^36,48^. Yeast strains were grown to saturation at 30 °C overnight, diluted 1:4 and then grown to log phase at 22 °C. Cell pellets (2.5 × 10^7^ cells) were washed and resuspended in H_2_O and incubated with Alkali/β-mercaptoethanol solution (1.85 M NaOH, 1.065 M β-mercaptoethanol) on ice for 10 min. TCA (50%) was then added and an additional 10 min incubation on ice was performed. Lysates were pelleted and resuspended in loading buffer (1X standard loading buffer, 1.42 M β-mercaptoethanol, 83.2 mM Tris–HCl, pH 8.8), boiled and re-pelleted. Supernatants were boiled at 95 °C for 5 min prior to gel loading.

### Western blot

Western blots were performed as previously described^36,49^. Total protein was separated on an 8% acrylamide gel and electrophoretically transferred to a nitrocellulose membrane. Ponceau staining ensured even transfer. Membranes were washed in 1X TBST and blocked with 5% milk, then washed with 1X TBST and incubated at 4 °C with primary antibodies (1:1000 for Actin and 1:5000 for Gag) overnight. Membranes were washed in 1X TBST, incubated with secondary antibodies (1:2000) for 1 h at RT, washed in 1X TBST, incubated with ECL substrate and digitally imaged using a ChemiDoc imager. Act1 is presented as a loading control. Uncropped blot images are included in Fig. S5.

### Sequential ChIP

Sequential chromatin immunoprecipitation was performed as previously described with slight modifications^50,51^. The first immunoprecipitation for RNA–DNA hybrids was performed as described above with the following exceptions: two IPs were performed per strain and two 15 min washes with elution buffer were performed at the elution step. After elution, the IPs were collected into a single 15 mL falcon tube per strain. A 100 µL aliquot of S9.6 IP was saved as input. Eluted samples were diluted 1:20 in lysis buffer (50 mM HEPES KOH, pH 7.5, 500 mM NaCl, 1 mM EDTA, 1% Triton X-100, 0.1% sodium deoxycholate, 0.1% SDS, 1 mM PMSF, Sigma cOmplete protease inhibitor) and incubated with 2 µg anti-ssDNA (Millipore MAB3034) overnight at 4 °C. Protein-A sepharose beads were added and incubation continued overnight at 4 °C. The beads were then washed in lysis buffer, followed by lithium chloride buffer (10 mM Tris–HCl, pH 8, 250 mM LiCl, 0.5% NP-40, 0.5% sodium deoxycholate, 1 mM EDTA) and TE (10 mM Tris–HCl, pH 8, 1 mM EDTA) to remove unbound DNA. Bound DNA was eluted from the beads (50 mM Tris–HCl, pH 8, 10 mM EDTA, and 1% SDS). Input and IP samples were incubated at 65 °C overnight to reverse crosslink. Samples were incubated at 37 °C for 30 min with 0.2 μg/μL RNase A, and for 2 h with 0.03 μg/μL glycogen and 0.2 μg/μL proteinase K to remove RNA and protein. DNA was isolated and analyzed by qPCR (1 cycle of 95 °C for 5 min, 60 °C for 30 s followed by 39 cycles of 95 °C for 5 s, 60 °C for 30 s and a final melt curve from 65 °C to 95 °C at 0.5 °C increments with 5 s/step). For qPCR analyses, ssDNA IP signals were divided by S9.6 input signals and normalized to CUP1 controls. For sequential ChIP data analysis, the first pulldown was conducted using the S9.6 antibody and the resulting pulldown samples were then used as input for a second pulldown with the ssDNA antibody. To assess how much ssDNA is present in every unit of S9.6 IP, we divided the signal from ssDNA pulldown over the signal obtained from the S9.6 pulldown. Additionally, the data were normalized to the ratios observed in wild-type cells, where low levels of Ty1-associated hybrids are known to exist as duplex structures related to the reverse transcription of Ty1 cDNA. A mutant with duplex Ty1 RNA-cDNA hybrid structures will resemble wild-type cells and have a ratio close to 1 (this is the case for *rnh1Δ rad27Δ*), while a mutant with triplex R-loop levels at Ty1 will have a value greater than the wild-type value of 1, since more ssDNA was pulled down per unit of S9.6 IP as compared to wild-type cells (this is the case for *rnh1Δ rnh201Δ*). Conversely, if the locus examined in wild-type cells were to contain mostly triplex R-loops (which is not the case for Ty1), a mutant with duplex RNA–DNA hybrids at that locus will have less ssDNA pulled down per unit of S9.6 IP as compared to wild-type (ratio < 1) while a mutant with triplex R-loop levels at that locus will resemble wild-type (ratio ~ 1). Overall, our data indicate that the Ty1 cDNA of wild-type cells and *rnh1Δ* *rad27Δ* cells are associated with duplex RNA–DNA hybrids while Ty1 in *rnh1Δ rnh201Δ* cells is associated with triplex R-loop structures. We note that normalization can be performed relative to wild-type cells or mutant cells, as long as the second pulldown with ssDNA provides a value greater than zero, which could be obtained in a very clean pulldown from cells where the locus examined does not harbour any triplex structures.

### Statistical analysis

Statistical analyses were performed using GraphPad Prism 7 and included two-tailed *t*-tests, one-way ANOVAs or their non-parametric equivalents where appropriate. Following ANOVAs, post-hoc analyses were carried out. Statistics are represented in the figures by asterisks and the corresponding statistical test is described in the figure legend. Results were considered significant if *p* < 0.05.

## Supplementary information

Supplementary file1

Supplementary file2

## Data Availability

The data supporting our findings are available in the article, supplementary information, and the source data file.
