# Supplementary material for: RNA-cDNA hybrids mediate transposition via different mechanisms
Source: Sci Rep. 2020 Sep 29;10:16034. doi: 10.1038/s41598-020-73018-y (PMC7524711; doi:10.1038/s41598-020-73018-y)
Supplement: Supplementary file 1 — Supplementary file1 [file 41598_2020_73018_MOESM1_ESM.pdf]

RNA-cDNA hybrids mediate transposition via different mechanisms

Lauren A. Todd[1,3], Amanda C. Hall[1,3], Violena Pietrobon[1], Janet N.Y. Chan[1], Guillaume Laflamme[1], and Karim Mekhail[1,2,*]

[1]*Department of Laboratory Medicine and Pathobiology, Faculty of Medicine, University of Toronto, Toronto, Ontario, M5G 1M1, Canada*

[2]*Canada Research Chairs Program, Faculty of Medicine, University of Toronto, Toronto Ontario, M5G 1M1, Canada*

[3]These authors contributed equally

*email: karim.mekhail@utoronto.ca

ORCID IDs:

L.A.T.: 0000-0001-6922-6196

A.C.H.: 0000-0002-2509-810X

V.P.: 0000-0001-7835-2223

J.N.Y.C.: 0000-0001-5644-985X

G.L.: 0000-0002-9083-4572

K.M.: 0000-0002-6084-020X

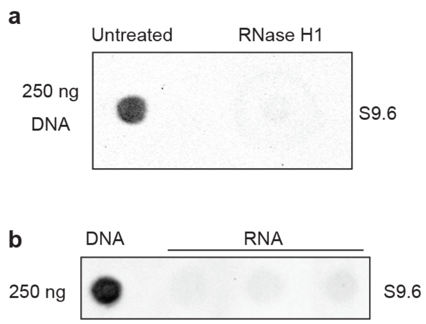

Fig. S1. Specificity of the anti-RNA-DNA hybrid S9.6 antibody. **(a)** Dot blot analysis confirms S9.6 signal sensitivity to RNase H1. Genomic DNA was incubated in the presence or absence of *in vitro* RNAse H1 treatment. 250 ng of DNA was spotted and crosslinked on a nylon membrane, blocked with 5% milk in 1X PBST, incubated with 1:5,000 S9.6 antibody (5% milk in 1X PBST) overnight at 4°C, incubated in 1:5,000 secondary antibody (5% milk in 1X PBST) and developed on a ChemiDoc using ECL substrate. **(b)** Dot blot analysis confirms the specificity of S9.6 to DNA-containing structures. 250 ng of total RNA was spotted and crosslinked and membranes were processed as described in (A).

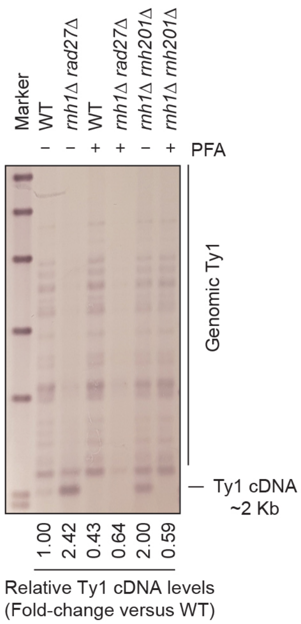

Fig. S2. Southern blot analysis of Ty1 cDNA levels following PFA treatment. These data serves as a second replicate of the Southern blot presented in Fig. 3c. M = marker.

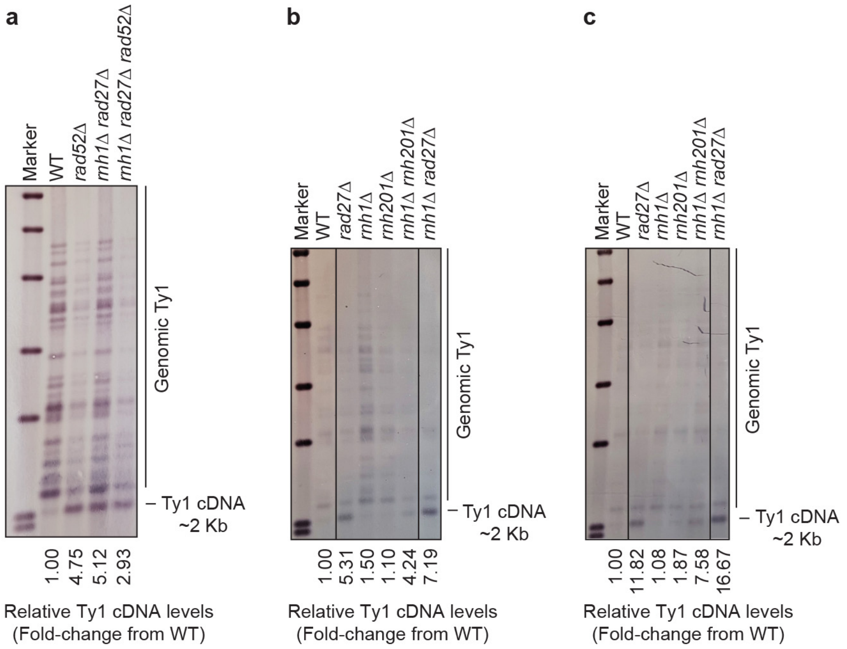

Fig. S3. Southern blot analyses of Ty1 cDNA levels in mutants of interest. (a) These data serve as a second replicate of the Southern blot presented in Fig. 5e. **(b)** Southern blot analysis comparing the levels of Ty1 cDNA in *rad27*Δ cells with that of *rnh1*Δ *rad27*Δ cells. **(c)** These data serve as the second replicate of the Southern blot presented in (b). **(a-c)** M = marker. Solid lines in **b** and **c** indicate where intervening lanes containing unrelated biological samples were cropped out and we note that all lanes shown in each of these two panels were run on the same gel and simultaneously exposed as shown in the corresponding uncropped blots in Fig. S5.

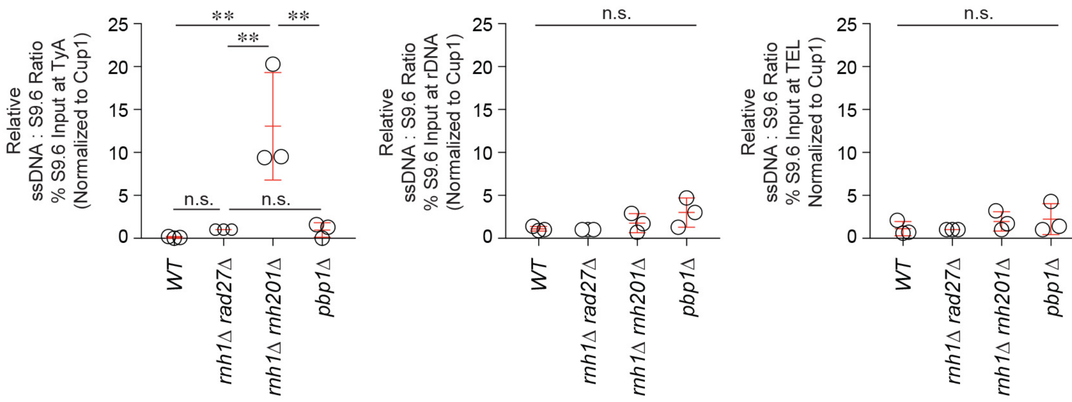

Fig. S4. Additional controls related sequential ChIPs. Graphs represent relative ssDNA/RNA-DNA hybrid ratios for cells with the indicated genotypes at TyA, ribosomal DNA (rDNA), and telomeres (TEL). Statistical significance was evaluated using a one-way ANOVA followed by Tukey's post-hoc test. Mean ± SD; $n = 3$. ** = $p < 0.01$ and n.s. = not statistically significant.

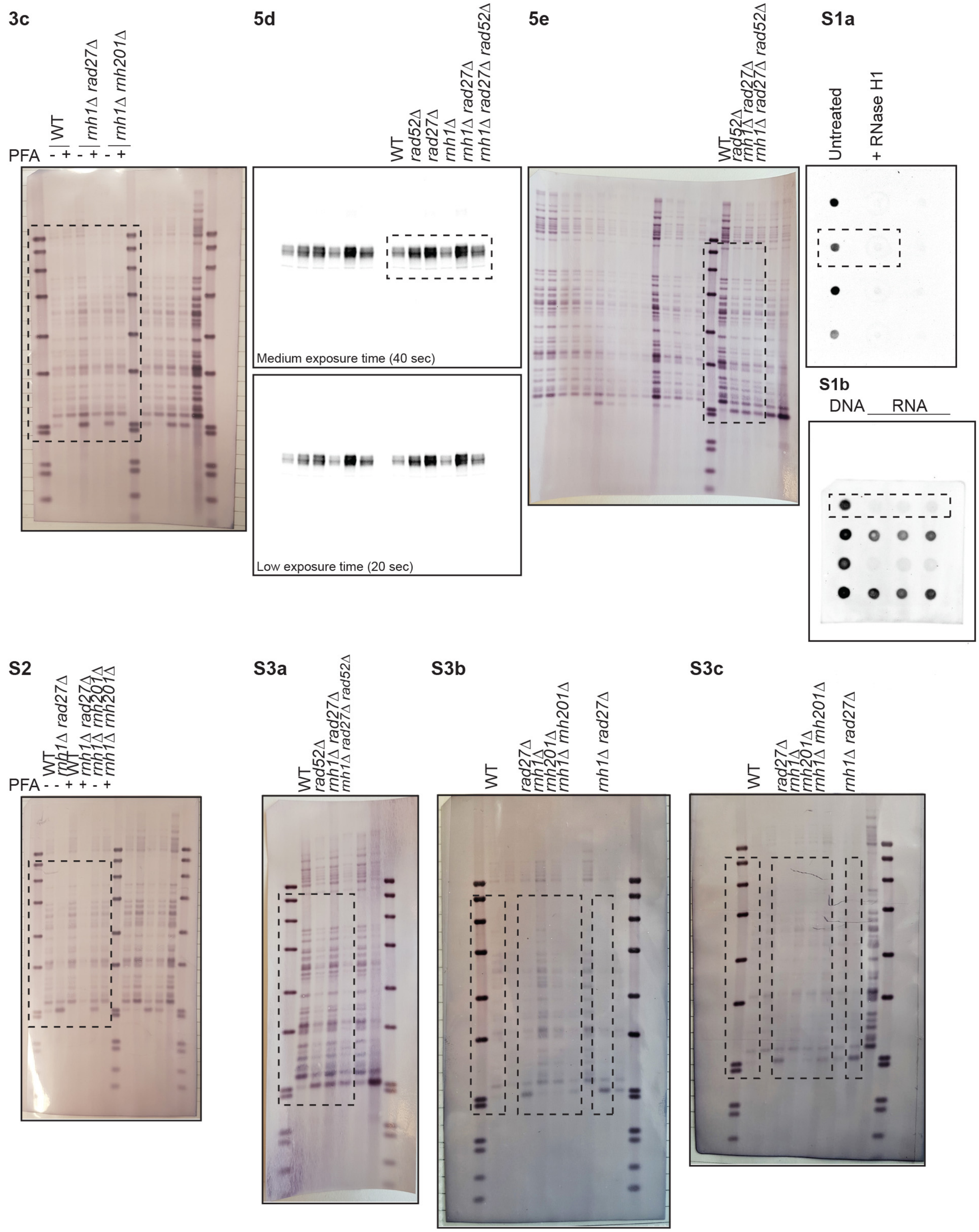

Fig. S5. Uncropped versions of all blots included in this study. The bolded numbers refer to the corresponding main figure panels and the dotted boxes outline the cropped area.

Table S1. Strains used in this study.

Strain	Genotype	Reference
KMY2372	Mat a his3Δ1, leu2Δ0, met15Δ0, ura3Δ0, Ty1his3AI[Δ1]-3114	JC3212
KMY2454	Mat a his3Δ1, leu2Δ0, met15Δ0, ura3Δ0, Ty1his3AI[Δ1]-3114, pbp1Δ::hphR	36
KMY2455	Mat a his3Δ1, leu2Δ0, met15Δ0, ura3Δ0, Ty1his3AI[Δ1]-3114, rad27Δ::hphR	36
KMY2494	Mat a his3Δ1, leu2Δ0, met15Δ0, ura3Δ0, Ty1his3AI[Δ1]-3114, rnh1Δ::kanR (clone 1)	This study
KMY2495	Mat a his3Δ1, leu2Δ0, met15Δ0, ura3Δ0, Ty1his3AI[Δ1]-3114, rnh1Δ::kanR (clone 2)	This study
KMY2496	Mat a his3Δ1, leu2Δ0, met15Δ0, ura3Δ0, Ty1his3AI[Δ1]-3114, pif1Δ::kanR	This study
KMY2557	Mat a his3Δ1, leu2Δ0, met15Δ0, ura3Δ0, Ty1his3AI[Δ1]-3114, rnh1Δ::kanR, rnh201Δ::hphR (clone 1)	36
KMY2558	Mat a his3Δ1, leu2Δ0, met15Δ0, ura3Δ0, Ty1his3AI[Δ1]-3114, rnh1Δ::kanR, rnh201Δ::hphR (clone 2)	36
KMY2571	Mat a his3Δ1, leu2Δ0, met15Δ0, ura3Δ0, Ty1his3AI[Δ1]-3114, pif1Δ::kanR, pbp1Δ::hphR	This study
KMY2573	Mat a his3Δ1, leu2Δ0, met15Δ0, ura3Δ0, Ty1his3AI[Δ1]-3114, rnh1::kanR, pbp1Δ::hphR	This study
KMY2577	Mat a his3Δ1, leu2Δ0, met15Δ0, ura3Δ0, Ty1his3AI[Δ1]-3114, rnh1::kanR, rad27Δ::hphR (clone 1)	This study
KMY2578	Mat a his3Δ1, leu2Δ0, met15Δ0, ura3Δ0, Ty1his3AI[Δ1]-3114, rnh1::kanR, rad27Δ::hphR (clone 2)	This study
KMY2581	Mat a his3Δ1, leu2Δ0, met15Δ0, ura3Δ0, Ty1his3AI[Δ1]-3114, pif1Δ::kanR, rad27Δ::hphR	This study
KMY2587	Mat a his3Δ1, leu2Δ0, met15Δ0, ura3Δ0, Ty1his3AI[Δ1]-3114, rnh201Δ::kanR (clone 1)	This study
KMY2588	Mat a his3Δ1, leu2Δ0, met15Δ0, ura3Δ0, Ty1his3AI[Δ1]-3114, rnh201Δ::kanR (clone 2)	This study
KMY2650	Mat a his3Δ1, leu2Δ0, met15Δ0, ura3Δ0, Ty1his3AI[Δ1]-3114, rnh201Δ::kanR, pbp1Δ::hphR	This study
KMY2653	Mat a his3Δ1, leu2Δ0, met15Δ0, ura3Δ0, Ty1his3AI[Δ1]-3114, rnh201Δ::kanR, pif1Δ::hphR	This study
KMY2734	Mat a his3Δ1, leu2Δ0, met15Δ0, ura3Δ0, Ty1his3AI[Δ1]-3114, rnh201Δ::kanR, rad27Δ::hphR	This study
KMY2735	Mat a his3Δ1, leu2Δ0, met15Δ0, ura3Δ0, Ty1his3AI[Δ1]-3114, rnh1::kanR, pif1Δ::hphR	This study
KMY2895	Mat a his3Δ1, leu2Δ0, met15Δ0, ura3Δ0, Ty1his3AI[Δ1]-3114, rnh1Δ::kanR rnh201Δ::hphR, rad52Δ::natR	This study
KMY3416	Mat a his3Δ1, leu2Δ0, met15Δ0, ura3Δ0, Ty1his3AI[Δ1]-3114, rad52Δ::URA (clone 1)	This study
KMY3417	Mat a his3Δ1, leu2Δ0, met15Δ0, ura3Δ0, Ty1his3AI[Δ1]-3114, rad52Δ::URA (clone 2)	This study
KMY3522	Mat a his3Δ1, leu2Δ0, met15Δ0, ura3Δ0, Ty1his3AI[Δ1]-3114, rnh1::kanR, rad27Δ:hphR, rad52Δ::URA (clone 1)	This study
KMY3523	Mat a his3Δ1, leu2Δ0, met15Δ0, ura3Δ0, Ty1his3AI[Δ1]-3114, rnh1::kanR, rad27Δ:hphR, rad52Δ::URA (clone 2)	This study
KMY3615	Mat a his3Δ1, leu2Δ0, met15Δ0, ura3Δ0, Ty1his3AI[Δ1]-3114, pRS416-URA Gal EV (clone 1)	This study
KMY3616	Mat a his3Δ1, leu2Δ0, met15Δ0, ura3Δ0, Ty1his3AI[Δ1]-3114, pRS416-URA Gal EV (clone 2)	This study
KMY3618	Mat a his3Δ1, leu2Δ0, met15Δ0, ura3Δ0, Ty1his3AI[Δ1]-3114, pRS416-URA Gal Rnh1 OE (clone 1)	This study
KMY3619	Mat a his3Δ1, leu2Δ0, met15Δ0, ura3Δ0, Ty1his3AI[Δ1]-3114, pRS416-URA Gal Rnh1 OE (clone 2)	This study
KMY3621	Mat a his3Δ1, leu2Δ0, met15Δ0, ura3Δ0, Ty1his3AI[Δ1]-3114, rnh1Δ::kanR rnh201Δ::hphR, pRS416-URA Gal EV (clone 1)	This study
KMY3622	Mat a his3Δ1, leu2Δ0, met15Δ0, ura3Δ0, Ty1his3AI[Δ1]-3114, rnh1Δ::kanR rnh201Δ::hphR, pRS416-URA Gal EV (clone 2)	This study
KMY3624	Mat a his3Δ1, leu2Δ0, met15Δ0, ura3Δ0, Ty1his3AI[Δ1]-3114, rnh1Δ::kanR	This study

	rnh201Δ::hphR, pRS416-URA Gal Rnh1 OE (clone 1)	
KMY3625	Mat a his3Δ1, leu2Δ0, met15Δ0, ura3Δ0, Ty1his3AI[Δ1]-3114, rnh1Δ::kanR rnh201Δ::hphR, pRS416-URA Gal Rnh1 OE (clone 2)	This study
KMY3627	Mat a his3Δ1, leu2Δ0, met15Δ0, ura3Δ0, Ty1his3AI[Δ1]-3114, rnh1::KanR, rad27Δ::hphR, pRS416-URA Gal EV (clone 1)	This study
KMY3628	Mat a his3Δ1, leu2Δ0, met15Δ0, ura3Δ0, Ty1his3AI[Δ1]-3114, rnh1::KanR, rad27Δ::hphR, pRS416-URA Gal EV (clone 2)	This study
KMY3630	Mat a his3Δ1, leu2Δ0, met15Δ0, ura3Δ0, Ty1his3AI[Δ1]-3114, rnh1::KanR, rad27Δ::hphR, pRS416-URA Gal Rnh1 OE (clone 1)	This study
KMY3631	Mat a his3Δ1, leu2Δ0, met15Δ0, ura3Δ0, Ty1his3AI[Δ1]-3114, rnh1::KanR, rad27Δ::hphR, pRS416-URA Gal Rnh1 OE (clone 2)	This study
KMY3667	Mat a his3Δ1, leu2Δ0, met15Δ0, ura3Δ0, Ty1his3AI[Δ1]-3114, rad27Δ::hphR, pRS416-URA Gal EV (clone 1)	This study
KMY3668	Mat a his3Δ1, leu2Δ0, met15Δ0, ura3Δ0, Ty1his3AI[Δ1]-3114, rad27Δ::hphR, pRS416-URA Gal EV (clone 2)	This study
KMY3670	Mat a his3Δ1, leu2Δ0, met15Δ0, ura3Δ0, Ty1his3AI[Δ1]-3114, rad27Δ::hphR, pRS416-URA Gal Rnh1 OE (clone 1)	This study
KMY3671	Mat a his3Δ1, leu2Δ0, met15Δ0, ura3Δ0, Ty1his3AI[Δ1]-3114, rad27Δ::hphR, pRS416-URA Gal Rnh1 OE (clone 2)	This study

Table S2. Oligonucleotides used in this study.

Name	Sequence	Reference
TYA-F	ATCTATGATTCCGTATACAC	36
TYA-R	AGGATGAATCAGTAAATGTA	36
TYB-F	AGAATACCGAGGAATCTATCATCGC	36
TYB-R	AGTCACCAATACCACCCAAACTG	36
Ty1His3-F	GGCCGTGCGTGGAGTAAAAA	36
Ty1His3-R	AAGAAAATGCGGGATCATCTC	36
Cup1-F	TGAAGGTCATGAGTGCCAAT	31
Cup1-R	TTCGTTTCATTTCCCAGAGCA	31
Act1-F	GCCTTCTACGTTTCCATCCA	31
Act1-R	GGCCAAATCGATTCTCAAAA	31
Ty1cDNA-F	GATACGATGAGGCAATCACC	20
Ty1cDNA-R	AGTCTCATTGCCTTTGTGCC	20
Rnh1-F	TAGTGGTATGAGTGCGCATG	31
Rnh1-R	CGTGCCGTTTCCAAAACTTG	31
rDNA-F	GGAAAGCGGGAAGGAATAAG	31
rDNA-R	CGATTCAGAAAAATTCGCACT	31
TEL-F	CATGACCAGTCCTCATTTCCATC	31
TEL-R	ACGTTTAGCTGAGTTTAACGGTG	31
